# ATRX Promotes Transcription Initiation of HSV-1 Immediate Early Genes During Early Lytic Infection

**DOI:** 10.3390/v17091169

**Published:** 2025-08-27

**Authors:** Laura E. M. Dunn, Mackenzie M. Clark, Joel D. Baines

**Affiliations:** Baker Institute for Animal Health, College of Veterinary Medicine, Cornell University, Ithaca, NY 14850, USA; led97@cornell.edu (L.E.M.D.);

**Keywords:** HSV-1, herpesviruses, transcription, initiation, PML, ATRX, G4

## Abstract

Herpes simplex virus 1 (HSV-1) transcribes its genome using host RNA polymerase II (Pol II) in a temporally regulated cascade. We previously proposed a model of Transient Immediate Early gene Mediated Repression (TIEMR), in which early repression of immediate early (IE) genes is relieved to initiate the cascade. Given the rapid association of promyelocytic leukaemia nuclear body (PML-NB) components with incoming HSV-1 genomes, we sought to investigate their roles in TIEMR. siRNA knockdown revealed that depletion of ATRX, but not PML, significantly reduced nascent transcription from viral IE promoters at 1.5 hpi, while DAXX knockdown increased transcription. ChIP-Seq showed ATRX localizes to both transcriptionally active IE genes and restricted non-IE genes, suggesting diverse functions. Notably, ATRX occupancy at active IE promoters correlated with G-quadruplex (G4) motifs, and G4 stabilization mimicked ATRX knockdown by reducing transcription initiation. These findings uncover a previously unrecognized pro-transcriptional role for ATRX at IE genes and suggest that ATRX promotes escape from TIEMR by facilitating transcription initiation and preventing G4-mediated repression.

## 1. Introduction

At the initiation of HSV-1 infection, histone-free viral genomes encoding more than 80 densely packed genes are delivered to the nucleus. These genomes contain a high concentration of promoter elements, including TATA boxes and initiator sequences recognized by cellular RNA Polymerase II (Pol II) [1,2]. As such, the viral genome is engaged by Pol II within minutes of infection [3,4]. However, the virus rapidly represses Pol II activity on its genome through the action of its IE genes in a process known as Transient Immediate Early gene Mediated Repression (TIEMR) [5]. Once this repression is established, transcription of viral genes is regulated by de-repression, leading to a temporal cascade of expression of different gene subsets. This begins with immediate–early (IE) genes, followed sequentially by early (E), leaky late (LL), and true late (L) genes [6,7].

The process of TIEMR also likely involves cellular proteins, as numerous nuclear factors interact with incoming HSV-1 DNA within the first hour of infection. These include proteins associated with transcriptional regulation, such as components of the NuRD and SWI/SNF chromatin remodelling complexes, as well as factors involved in the DNA damage response [8]. Several studies have also shown that nuclear HSV-1 genomes rapidly colocalize with Promyelocytic Leukaemia Nuclear Bodies (PML-NBs) [8,9,10,11]. PML-NBs are multi-protein nuclear domains containing core components PML, sp100, ATRX, and DAXX [11,12,13]. The sequestration of viral DNA in PML-NBs is assumed to be an intrinsic immune response to repress HSV-1 infection [14,15,16]. HSV-1 counteracts this response through the PML-NB antagonist, ICP0, which acts as a RING-finger ubiquitin ligase to promote the degradation of PML, resulting in the dissolution of PML bodies [17,18].

The requirement for early transcriptional repression on the viral genome [5] led us to hypothesize that the virus exploits PML-NB-associated repression to facilitate the establishment of TIEMR. Here, we demonstrate that while DAXX does contribute to early transcription repression, we unexpectedly found that its binding partner [19] ATRX promotes transcription initiation on viral IE genes at 1.5 hpi. ATRX has traditionally been regarded as a repressor due to its stabilizing H3.3 on the viral genome, which can be linked to heterochromatin formation [13,20]. However, using a ChIP-Seq protocol that improves the detection of proteins such as ATRX that bind DNA indirectly [21], we show that, as on different cellular genes, ATRX localizes not only to restricted transcription regions but also actively transcribing sites, suggesting a more complex and diverse role in ATRX regulation of viral gene expression than previously appreciated. Furthermore, we provide evidence supporting the hypothesis that ATRX-mediated transcriptional activation is facilitated by its association with G-quadruplex (G4) DNA structures at viral IE promoters.

## 2. Materials and Methods

### 2.1. Cells

HEp-2 (human epithelial lung cancer, CCL-23) and Vero (African green monkey, CCL-81) cells were both obtained from ATCC, Manassas, VA, USA) and maintained in Dulbecco’s modified Eagle’s medium (DMEM, Gibco, Waltham, MA, USA) containing 10% new-born calf serum (NBS, Sigma Aldrich, Saint Louis, MO, USA), 100 units/mL penicillin, 100 μg/mL streptomycin (pen/strep, Gibco, Waltham, MA, USA) and maintained at 37 °C with 5% CO_2_. HFF (hTERT immortalized human foreskin fibroblasts, BJ-5ta) cells were acquired from ATCC (Manassas, VA, USA) and maintained in 4:1 DMEM:199 V medium (Gibco, Waltham, MA, USA) supplemented with 10% Foetal bovine serum (FBS, Corning, Tewksbury, MA, USA), pen/step and 0.01 mg/mL hygromycin B (Gibco, Waltham, MA, USA).

### 2.2. siRNA Knockdown

Dicer-Substrate siRNAs were ordered from Integrated DNA Technologies (IDT, Coralville, IA, USA) [22] for PML (hs.Ri.PML.13.1, #452537617), DAXX (hs.Ri.DAXX.13.1, #467654972), ATRX (hs.Ri.ATRX.13.1, #452537611) and non-targeting negative control (DS NC1, #51-01-14-04). An amount of 50 nM siRNA was reverse transfected using Lipofectamine RNAimax reagent (Thermo Fisher, Waltham, MA, USA), and cells were seeded on top in a pen/strep-free medium.

### 2.3. mRNA RT-qPCR

RNA was extracted from whole cell extracts using Monarch Spin RNA Mini Kit (NEB, Ipswich, MA, USA). Reverse transcription was performed using SuperScript III primed with Oligo(dT) (Invitrogen, Waltham, MA, USA). qPCR analysis of the cDNA was performed using Luna Universal qPCR Master Mix (NEB). Primers are listed in Table S1. Relative expression to the negative control knockdown was calculated using the *ΔΔCt* method as below:

$$\Delta Ct=Ct\left(gene\;of\;interest\right)-Ct\;\left(SDHA\right)$$

$$\Delta \Delta Ct=\Delta Ct-\Delta Ct\left(mean\;Neg\;ctrl\;siRNA\right)$$

$${\;2}^{-\Delta \Delta Ct}=relative\;expression$$

### 2.4. Viruses, Infection, and Drug Treatment

HSV-1(F) stocks were prepared and titered on Vero cells. Monolayers of HEp-2 cells were infected with HSV-1 at an MOI of 5 in 199 V medium supplemented with 1% NBS and pen/strep. Cells were incubated at 4 °C for 1 h to allow for virus adsorption. After 1 h, the inoculum was removed and replaced with pre-warmed (37 °C) DMEM with 2% serum, and infection was allowed to proceed. This was time 0 hpi. For BRACO-19 treatment, 25 μM (or an equal volume of DMSO (Thermo Fisher, Waltham, MA, USA)) was added to the pre-warmed media at 0 hpi. This concentration was chosen based on previous studies, which indicated that this was non-cytotoxic [23,24,25].

### 2.5. Viral Yield Assay

Monolayers of siRNA-treated HEp-2 cells were infected with HSV-1 at an MOI of 5 in 199 V medium (plus 1% NBS) at 4 °C. After 1 h, inoculum was removed and replaced with pre-warmed DMEM (with 2% NBS). Infected cells were harvested at 24 hpi by scraping into media, and viruses were released from cells by 3× cycles of freeze-thaw in LN_2_ and 37 °C water bath. Virus yields were determined by plaque assay on Vero cells.

### 2.6. Nuclei Isolation

Nuclei were isolated from infected using cells as previously described [26,27]. For siRNA knockdowns, 2 × 10^6^ cells were used per replicate; for all other experiments, 8 × 10^6^ cells were used. Cells were washed 2x with ice-cold PBS incubated for 10 min on ice with swelling buffer (10 mM Tris-HCl [pH 7.5], 10% glycerol, 3 mM CaCl_2_, 2 mM, MgCl_2_, 0.5 mM DTT, protease inhibitors [Pierce] and 4 U/mL RNase inhibitors [RNaseOUT, ThermoFisher]). Cells were scraped from the plate, pelleted via centrifugation at 600× *g* for 10 min (4 °C), resuspended in lysis buffer (swelling buffer + 0.5% Igepal (Sigma Aldrich, Saint Louis, MO, USA)), and then incubated on ice for 20 min to release nuclei. Nuclei were pelleted by centrifugation at 1500× *g* for 5 min (4 °C), washed 2x with lysis buffer with a final wash in storage buffer (50 mM Tris-HCl [pH 8.0], 25% glycerol, 5 mM MgAcetate, 0.1 mM EDTA, 5 mM DTT). Nuclei were resuspended in a storage buffer, flash-frozen in LN_2_, and stored at −80 °C.

### 2.7. Nuclear Run-On

For RT-qPCR, frozen nuclei were thawed on ice and a 2-biotin run-on performed by addition of an equal volume of run-on buffer (10 mM Tris-HCl [pH 8.0], 5 mM MgCl_2_, 1 mM DTT, 300 mM KCl, 200 μM ATP, 200 μM UTP, 200 μM biotin-11-GTP, 200 μM biotin-11-CTP, 0.4 U/mL RNase inhibitor and 1% Sarkosyl). Run-on was performed under constant shaking for 5 min on a vortex shaker at 37 °C.

For PRO-Seq, frozen nuclei were thawed on ice and a 4-biotin run-on was performed as previously detailed [26,27]. An equal volume of run-on buffer (10 mM Tris-HCl [pH 8.0], 5 mM MgCl_2_, 1 mM DTT, 300 mM KCl, 20 μM biotin-11-ATP, 20 μM biotin-11-GTP, 200 μM biotin-11-CTP, 20 μM UTP, 0.4 U/mL RNase inhibitor and 1% Sarkosyl) was added to thawed nuclei. Run-on was performed under constant shaking for 3 min on a vortex shaker at 37 °C.

All run-on reactions were ended by adding TRIzol LS (Invitrogen, Waltham, MA, USA) and RNA extracted following the manufacturer’s protocol (MAN0000806).

### 2.8. PRO-RTqPCR

Extracted RNA was subjected to sequential enzyme digestion to ensure the removal of viral DNA, first with XbaI for 2 h at 37 °C and then with TURBO DNase (Invitrogen, Waltham, MA, USA) overnight at 37 °C. RNA was cleaned-up using the Monarch Spin RNA Cleanup Kit (NEB, Ipswich, MA, USA) and biotinylated RNA was purified using streptavidin M280 Dynabeads (Invitrogen, Waltham, MA, USA), washed 1x in ice-cold high salt wash (50 mM Tris-HCl [pH 7.4], 2 M NaCl, 0.5% Triton X-100, 0.4 U/mL RNase inhibitor), 1x in ice-cold medium salt wash (10 mM Tris-HCl [pH 7.4], 300 mM NaCl, 0.1% Triton X-100, 0.4 U/mL RNase inhibitor) and 1x in ice-cold low salt wash (5 mM Tris-HCl [pH 7.4], 0.1% Triton X-100, 0.4 U/mL RNase inhibitor). RNA was eluted from beads via TRIzol extraction and reverse transcribed using SuperScript III primed with random hexamers. qPCR analysis of the cDNA was performed using Luna Universal qPCR Master Mix (NEB, Ipswich, MA, USA). Primers are listed in Table S1. Relative run-on activity to the negative control knockdown was calculated using the *ΔΔCt* method as below:

$$\Delta Ct=Ct\left(gene\;of\;interest\right)-Ct\;\left(ACTB\right)$$

$$\Delta \Delta Ct=\Delta Ct-\Delta Ct\left(mean\;Neg\;ctrl\;siRNA\right)$$

$$2^{-\Delta \Delta Ct}\times genome\;copy\;norm\;factor\;=\;\mathrm{relative}\;\mathrm{run-on}\;\mathrm{activity}$$

### 2.9. PRO-Seq Library Preparation

Libraries were prepared following a modified, rapid PRO-Seq protocol [28]. RNA was extracted from run-on nuclei via TRIZOL extraction and subjected to base hydrolysis for 10 min on ice with 0.2 N NaOH. Unincorporated nucleotides were removed through buffer exchange in a P-30 column (Bio-Rad, Hercules, CA, USA). As described above, biotinylated RNA was purified using streptavidin M280 Dynabeads. The 3′-RNA adapter (RA5) was ligated to the 3′ end of the RNA using T4 RNA ssRNA ligase I (NEB, Ipswich, MA, USA).

Biotinylated RNA was again bound to streptavidin M280 beads and 5′ cap removal with 10 U of 5′-pyrophosphohydrolase (RppH) (NEB, Ipswich, MA, USA) and 5′ end repair with T4 PNK (NEB, Ipswich, MA, USA) was performed on beads for 1 h at 37 °C. Beads were washed 1x high salt, 1x low salt, 1x DEPC H_2_O between enzyme incubation steps. Ligation of the RA3 5′-RNA adapter was performed on beads using T4 RNA ssRNA ligase I. RNA was eluted from beads via TRIzol extraction and reverse transcribed with SuperScript III using RNA PCR primer 1. The cDNA was PCR amplified with Phusion high-fidelity DNA Polymerase (NEB, Ipswich, MA, USA) using barcoded Illumina PCR index primers. Libraries were purified on an 8% polyacrylamide-TBE gel and underwent paired-end sequencing on an Illumina NovaSeq X Plus (2 × 150 bp) (Novogene, Sacramento, CA, USA).

### 2.10. PRO-Seq Data Analysis

FASTQ files were processed using the PRO-Seq 2.0 pipeline, developed by the Danko lab at Cornell: https://github.com/Danko-Lab/proseq2.0. The genome used to align reads was a concatenated file containing hg38 and HSV-1 F genomes. The HSV-1 F genome build had the external repeats deleted to aid sequencing alignment; the modified HSV-1 F genome file is available: https://github.com/Baines-Lab/Public/tree/main/HSV-1. Data were normalized for sequencing depth based on total paired reads and viral genome copy number, detailed in Table S3. HSV-1 normalized bigwig files were visualized using IGV genome browser [29].

### 2.11. Viral Genome Copy Number Quantification

After nuclear run-on, TRIzol LS RNA extraction, the lower interphase, and organic layers were saved. The DNA was subsequently isolated following the manufacturer’s protocol for TRIzol DNA isolation (Invitrogen, MAN0000806). DNA was cleaned up by 2x phenol:chloroform:isoamyl alcohol purification, followed by ethanol/sodium acetate precipitation and resuspended in DEPC H_2_O (Invitrogen, Waltham, MA, USA). DNA was quantified using Qubit high-sensitivity dsDNA kit. 5 ng of DNA was used as input, and qPCR performed using Luna Universal qPCR Master Mix (NEB, Ipswich, MA, USA) with UL51 primers (sequences in Table S1). Genome copy number was determined by standard curve for UL51 as previously described [27,30]. The genome copy norm factor was calculated as below:

(mean all samples genome copy number)/(sample genome copy number)

### 2.12. Immunofluorescence and Confocal Microscopy

siRNA transfected HEp-2 cells seeded onto 13 mm glass coverslips were fixed in 4% paraformaldehyde (PFA) at 1.5 hpi for 15 min, washed in 3XPBS and permeabilized by adding 0.2% Triton X-100 diluted in PBS for 10 min. Cells were washed 3XPBS and blocked for 1 h at room temperature in 2% donkey serum (DS) diluted in PBS. Primary antibodies were diluted in PBS + 2% DS and incubated with the cells in a humidity chamber for 1 h. Cells were washed 3x in PBS and incubated with secondary antibodies diluted in PBS + 2% DS in a humidity chamber for 1 h. Cells were washed 3x in PBS, stained with DAPI for 5 min, rinsed 3x in PBS with a final rinse in distilled H_2_O, and mounted onto a microscope slide using ProLong Glass Antifade Mountant (Invitrogen, Waltham, MA, USA). All incubation steps were performed at room temperature. The details of the antibodies used are in Table S2.

1024 × 1024 images were acquired on an Olympus (Waltham, MA, USA) Fluoview FV3000 laser scanning confocal microscope using the 60x oil immersion lens. Images were processed in ImageJ Fiji (Version: 2.14.0.154f). A custom CellProfiler (Version 4.2.6) [31] pipeline was developed to count nuclear foci in processed images.

### 2.13. Chromatin Immunoprecipitation and Sequencing (ChIP-Seq)

ATRX-ChIP was performed following the optimized protocol described in [21]. In brief, 10^8^ HSV-1 infected (MOI: 5) HEp-2 cells were resuspended in PBS at 1.5 hpi and crosslinked with 2 mM EGS (Thermo Scientific, Waltham, MA, USA) for 45 min at room temperature, followed by a 1% formaldehyde fixation for 8 min, then quenched in 125 mM glycine. Cells were washed 3x in ice-cold PBS and lysed sequentially, first with lysis buffer 1 (100 mM HEPES, 140 mM NaCl, 1 mM EDTA, 10% glycerol, 0.5% NP-40 and 0.25% Triton X-100), then with lysis buffer 2 (200 mM NaCl, 1 mM EDTA, 0.5 mM EGTA and 10 mM Tris pH 8) and finally with lysis buffer 3 (1 mM EDTA, 0.5 mM EGTA, 1 mM Tris–HCl pH 8, 100 mM NaCl, 0.1% sodium deoxycholate and 0.5% N-lauroyl sarcosine). All lysis buffers also contained protease inhibitors (Pierce, Thermo Scientific, Waltham, MA, USA). Samples were sheared with a G27 needle before sonication in a Bioruptor Plus (Diagenode, Denville, NJ, USA) (45 cycles of 30 s on/30 s off, 4 °C). Sonicated chromatin was pre-cleared with Protein A Dynabeads (Invitrogen, Waltham, MA, USA)) for 1 h at 4 °C, and 5% (vol) of the sample was saved as input. The remaining sample was split into two and incubated with either Protein A Dynabeads pre-coated with 10 μg anti-ATRX antibody (#ab97508, Abcam, Waltham, MA, USA) or 10 μg IgG (Abcam (#ab171870)) overnight at 4 °C. Beads were washed 5x in wash buffer (50 mM HEPES, 1 mM EDTA, 0.7% sodium deoxycholate, 500 mM LiCl) and bound chromatin eluted with 0.5% SDS and 100 mM sodium bicarbonate. DNA was reverse crosslinked in 0.2 M NaCl at 65 °C overnight and extracted by phenol:chloroform:isoamyl alcohol purification, followed by ethanol/sodium acetate precipitation and resuspended in DEPC H_2_O.

Sequencing libraries were prepared using NEBNext Ultra II DNA Library Prep Kit for Illumina (#E7645S, NEB, Ipswich, MA, USA), without size selection, following the manufacturer’s protocol. Libraries underwent paired-end sequencing on an Illumina NovaSeq X Plus (2 × 150 bp) (Novogene, Sacramento, CA, USA).

### 2.14. ChIP-Seq Data Analysis

FASTQ files were processed using a custom pipeline (https://github.com/Baines-Lab/Public/blob/main/ATRX_ChIP_Seq/QC_align.sh) to the same concatenated genome as used in PRO-Seq (hg38 and HSV-1 F). The HSV-1 reads were extracted from the .bam files and normalized using DeepTools [32] bamCoverage with RPGC normalization (--effectiveGenomeSize 136446) from RPGC normalization of hg38 reads (--effectiveGenomeSize 2913022398) to generate bigwig files. Peaks were called using MACS3 [33]. The bdgdiff command was used to call peaks on the HSV-1 genome (https://github.com/Baines-Lab/Public/blob/main/ATRX_ChIP_Seq/MACS3.sh). HOMER findMotifsGeneome.pl [34] was used to identify enriched motifs in ATRX peaks at IE genes using non-IE peaks as background.

### 2.15. Statistical Analysis

Statistical analysis was performed in R (version 4.4.2). Details of statistical tests and *p*-values are detailed throughout.

## 3. Results

### 3.1. ATRX Depletion Reduces Transcriptional Activity on HSV-1 Genes at 1.5 Hpi

**Figure 1:**
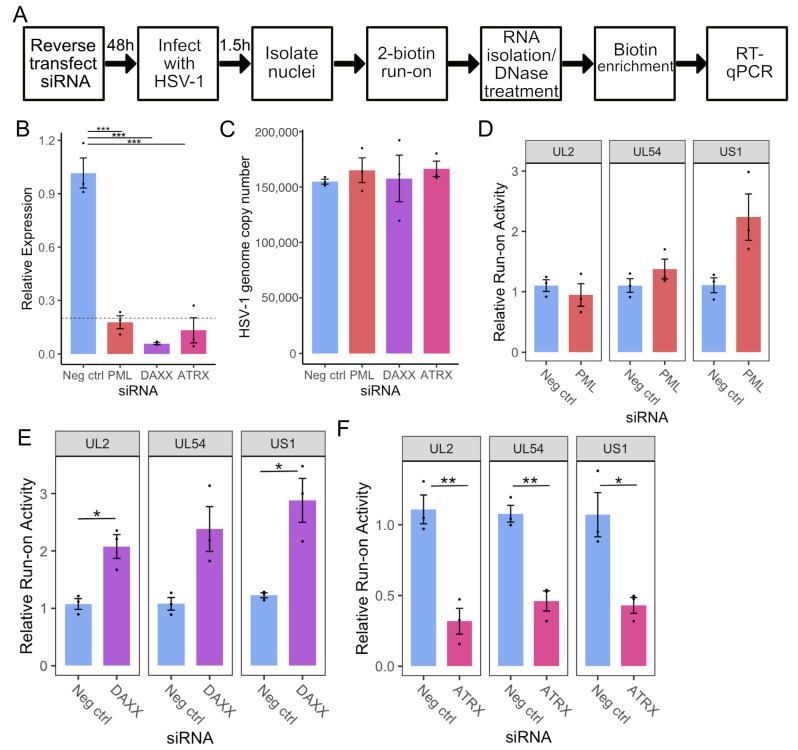
**ATRX depletion reduces transcriptional activity on HSV-1 genes at 1.5 hpi**. (**A**) Overview of siRNA KD coupled to RT-qPCR methodology for nascent RNA analysis. (**B**) RT-qPCR validation of siRNA knockdown at the mRNA level. Data are mean ± standard error. Plotted values are relative to the average negative control (non-targeting), normalized to SDHA. Statistical significance was determined using Dunnett’s test, with neg ctrl as the control group. (**C**) HSV-1 genome copy per 5 ng of DNA in siRNA knockdown at 1.5 hpi, determined by *UL51* plasmid standard curve qPCR. Data are mean ± standard error. Nascent transcriptional activity of HSV-1 genes at 1.5 hpi during PML knockdown (**D**), DAXX knockdown (**E**), and ATRX knockdown (**F**). Data are mean ± standard error. Plotted values are relative to the average negative control (non-targeting), normalized to ACTB and viral genome copy number. Statistical significance was determined using Welch’s *t*-test. Asterisks indicate statistical significance (* = *p* < 0.05, ** = *p* < 0.01, *** = *p* < 0.001).

To dissect the roles of individual PML-NB constituents in regulating early HSV-1 transcription, we first developed a system to screen for the effects of gene knockdowns on nascent transcription. Previous work in our laboratory relied on nuclear run-on assays coupled with sequencing [3,30]. Though this method provides unparalleled detail of Pol II activity, it is time-consuming and requires a large amount of starting material (0.5–2 × 10^7^ cells), making the protocol unsuitable for screening. We adapted an RT-qPCR-based method [35] to address this challenge by incorporating biotin-NTP run-on and streptavidin pulldown of nascent RNA. HSV-specific primers were then used for RT-qPCR to assess transcriptional activity at specific regions on the HSV-1 genome, normalized to the human gene ACTB (validated as a run-on reference gene by Roberts et al. [35]). An overview of this protocol is shown in Figure 1A. Primers were designed to amplify regions known to have detectable levels of nascent transcription on the viral genome at 1.5 h post-infection (hpi) [5], (Listed in Table S1).

Knockdown was achieved through siRNA transfection of HEp-2 cells, targeting the PML-NB constituent genes PML, DAXX, and ATRX alongside a non-targeting negative control. After 48 h, cells were infected with HSV-1 (F) at an MOI of 5, and nuclei were harvested at 1.5 hpi. RT-qPCR confirmed at least 80% knockdown at the mRNA level of each target gene relative to the negative control (Figure 1B). Nuclear run-on was then performed, and RT-qPCR was used to quantify nascent transcription of *UL2, UL54* (*α27*), and US1(*α22*) on the viral genome. The run-on qPCR data was additionally corrected by viral genome copy number, which was determined via qPCR on DNA isolated from the respective samples. HSV-1 genome copy results are shown in Figure 1C, indicating only minor variations in copies of input viral genomes among siRNA treatments.

Depletion of PML had a minimal effect on viral transcription, with only a detectable increase on *US1* that did not reach statistical significance (Figure 1D). Depletion of DAXX led to an approximately 2-fold increase in transcriptional activity at all regions analyzed (Figure 1E). In contrast, depletion of DAXX’s binding partner, ATRX, resulted in an approximately 2-fold decrease in transcription of all genes tested (Figure 1F). We confirmed these changes in nascent transcription during ATRX or DAXX depletion in TERT-immortalized human foreskin fibroblasts (HFFs). As in HEp2 cells, DAXX knockdown in HFFs increased *UL2, UL54*, and *US1* transcription at 1.5 hpi, whereas ATRX knockdown reduced transcription of these genes (Figure S1). Thus, it is apparent that ATRX and DAXX have distinct roles in early transcriptional regulation and that ATRX promotes transcription upon initial infection.

### 3.2. PML-NBs Retain the Ability to Form in ATRX-Depleted Cells

The finding that ATRX plays a pro-transcriptional role was interesting because this contrasts with its assumed role as a restriction factor [36]. We, therefore, sought to characterize this activity in more depth using our siRNA system. First, RT-qPCR confirmed that the loss of nascent transcription also reduced HSV-1 mRNA (Figure S2A). As DAXX and ATRX are binding partners [19], we also assessed DAXX expression by RT-qPCR during ATRX KD, which revealed no significant effect on DAXX mRNA levels (Figure S2B).

**Figure 2:**
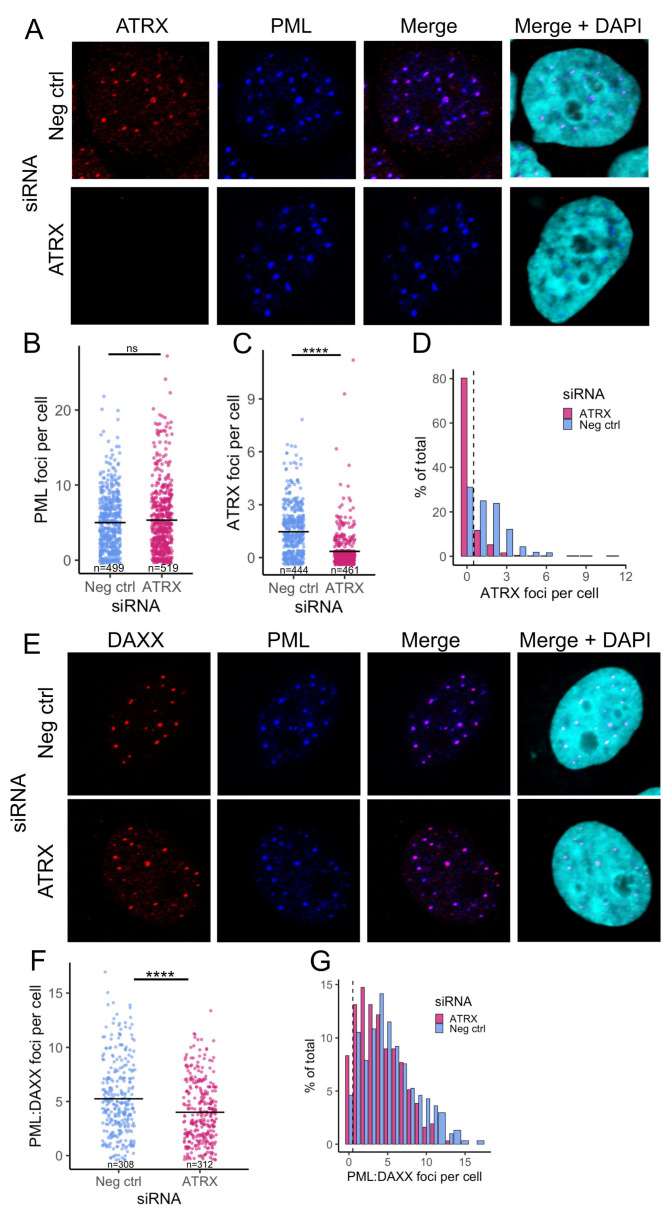
**PML-NBs retain the ability to form in ATRX-depleted cells**. (**A**) Representative confocal images of ATRX (red) and PML (blue) expression after 48 h ATRX siRNA knockdown, compared to neg ctrl (non-targeting) knockdown. Nuclei were stained with DAPI. Quantification of PML (**B**) and ATRX (**C**) foci per cell (nucleus). Each dot represents an individual cell. n = number of nuclei counted per condition. (**D**) Histogram of the frequency of ATRX foci counts. (**E**) Representative confocal images of DAXX (red) and PML (blue) expression after 48 h ATRX siRNA knockdown, compared to neg ctrl (non-targeting) knockdown. Nuclei were stained with DAPI. (**F**) Quantification of the PML:DAXX colocalized foci per cell. Each dot represents an individual cell. n = number of nuclei counted per condition. (**G**) Histogram of the frequency of PML:DAXX foci counts. Statistical significance was determined using Welch’s *t*-test. Asterisks indicate statistical significance (**** = *p* < 0.0001, ns = non-significant).

Next, we analyzed the ability of PML-NBs to form in the absence of ATRX to ensure the effect was not caused indirectly by the perturbation of PML-NBs. ATRX-depleted cells were stained for both PML and ATRX by immunofluorescence. PML foci were distinct in the Neg ctrl and showed strong colocalization with ATRX foci (Figure 2A, upper panel). While the ATRX KD cells displayed distinct PML foci, they lacked detectable ATRX foci, as expected (Figure 2A, lower panel). CellProfiler software [31] was then used to quantify these observations. ATRX KD did not significantly affect the PML foci counted per cell (Figure 2B) but confirmed a significant depletion in ATRX foci per cell (Figure 2C). In addition, over 80% of the PML-positive cells from ATRX KD contained no detectable ATRX foci (Figure 2D), further indicating that the siRNA knockdown of ATRX mRNA conferred a decrease in ATRX protein in PML-NB’s.

The unchanged total PML foci per cell in ATRX KD indicated that PML-NBs still form. To further validate this, we also stained for DAXX, another major component of PML-NBs and a binding partner of ATRX. Both PML and DAXX foci were readily detectable. They appeared to colocalize in both ATRX KD and Neg ctrl (Figure 2E). CellProfiler was then used to quantify the number of DAXX foci per cell (Figure S2D) and those that colocalized with PML. This revealed a slight drop in DAXX-PML-associated NBs during ATRX KD, with the mean number dropping to 4 per cell from 5.25 in the Neg ctrl (Figure 2F).

Plotting the distribution of DAXX/PML foci highlighted the trend toward a lower number of foci per cell during ATRX depletion. However, over 91% of cells still contained DAXX-PML-associated NBs (Figure 2G). In addition, we saw no evidence of an increase in these repressive factors (DAXX and PML) that might contribute to the transcriptional reduction that occurs during ATRX depletion. Together, these data indicate that the transcriptional effects of ATRX knockdown are ATRX-specific and not a result of alteration of other PML-NB components.

### 3.3. ATRX Promotes Transcription Initiation on IE Genes at 1.5 Hpi

Next, we used PRO-Seq to characterize II activity across the viral genome in ATRX-depleted cells. Unlike Pol II ChIP-Seq, which relies on antibody specificity to infer transcriptional activity indirectly by the location of DNA binding, PRO-Seq directly captures nascent RNA at nucleotide resolution, providing a precise and unbiased readout of active transcription [37]. This technique involves a nuclear run-on using biotin-labelled nucleotides, enabling the selective capture of nascent transcripts without poly(A) selection. As a result, PRO-Seq can detect rapid, transient, or short non-polyadenylated RNAs that are typically missed by conventional RNA-Seq methods.

**Figure 3:**
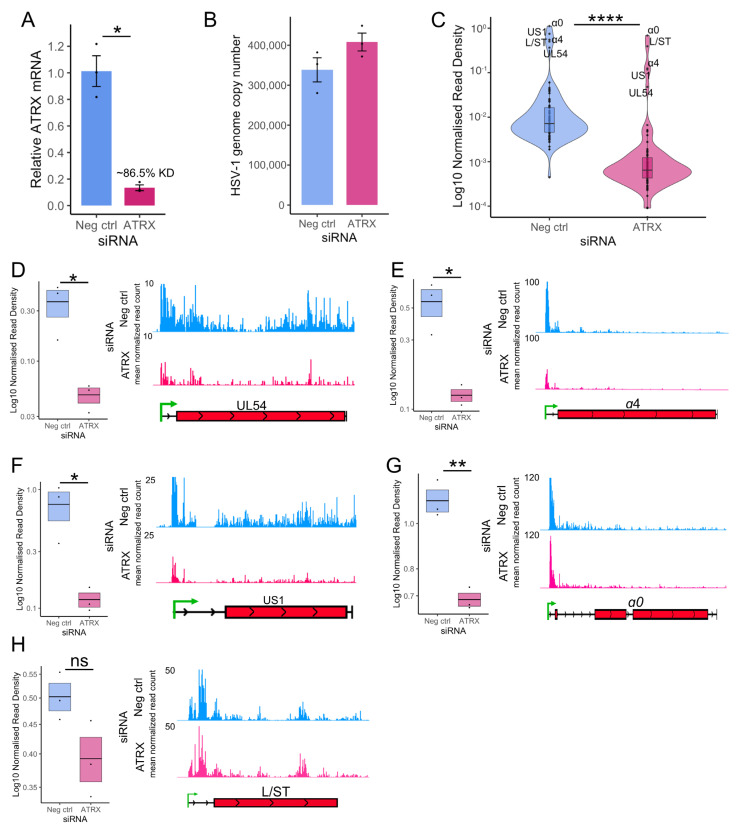
**ATRX promotes transcription on IE genes at 1.5 hpi**. (**A**) RT-qPCR validation of ATRX knockdown at the mRNA level in cells used for PRO-Seq. Data are mean ± standard error. Plotted values are relative to the average negative control (non-targeting), normalized to SDHA. (**B**) HSV-1 genome copy per 5 ng of DNA in siRNA knockdown at 1.5 hpi, determined by UL51 plasmid standard curve qPCR. Data are mean ± standard error. (**C**) PRO-Seq read density across viral genes at 1.5 hpi during ATRX knockdown, compared to neg ctrl (non-targeting) knockdown. Statistical significance was determined using the Wilcoxon test. PRO-Seq read density of individual IE genes and genome browser tracks at 1.5 hpi of (**D**) *UL54*, (**E**) *α4*, (**F**) *US1*, (**G**) *α0* and (**H**) *L/ST*. PRO-Seq read density is calculated as read per bp, normalized to spike-in and HSV-1 genome copy. Black lines indicate mean and bands ± standard error. Statistical significance was determined using Welch’s *t*-test. Asterisks indicate statistical significance (* = *p* < 0.05, ** = *p* < 0.01, **** = *p* < 0.0001, ns = non-significant).

HEp-2 cells were transfected with siRNA for 48 h, followed by infection with HSV-1 at an MOI of 5 PFU/cell. After 1 h adsorption at 4 °C, infection was allowed to proceed at 37 °C. Nuclei were then harvested at 1.5 hpi, and PRO-Seq was performed. Knockdown was validated by RT-qPCR (Figure 3A), and qPCR was used to assess input viral genome copy in the run-on nuclei, confirming there was no significant difference between groups (Figure 3B). Noted minor differences in genome copy number were accounted for in normalization of the PRO-Seq data, which was also corrected to the total library read count to account for variation in sequencing depth (details in Table S3). Calculation of the density of reads across each HSV-1 gene indicated that ATRX depletion led to a significant reduction in transcriptional activity of all viral genes at 1.5 hpi (Figure 3C), confirming the result from the prior qPCR run-on assay (Figure 1F).

Next, we focused the analysis on the IE genes, the most critical genes at 1.5 hpi and the most robustly transcribed (Figure 3C). The PRO-Seq read density of each IE gene was calculated and analyzed, alongside IGV genome browser displays for visualization (Figure 3D–G). This confirmed a significant decrease in transcriptional activity across all IE genes. *L/ST* was also analyzed because it was highly transcribed at 1.5 hpi. Transcriptional activity across *L/ST* was not significantly altered by ATRX depletion (Figure 3H).

**Figure 4:**
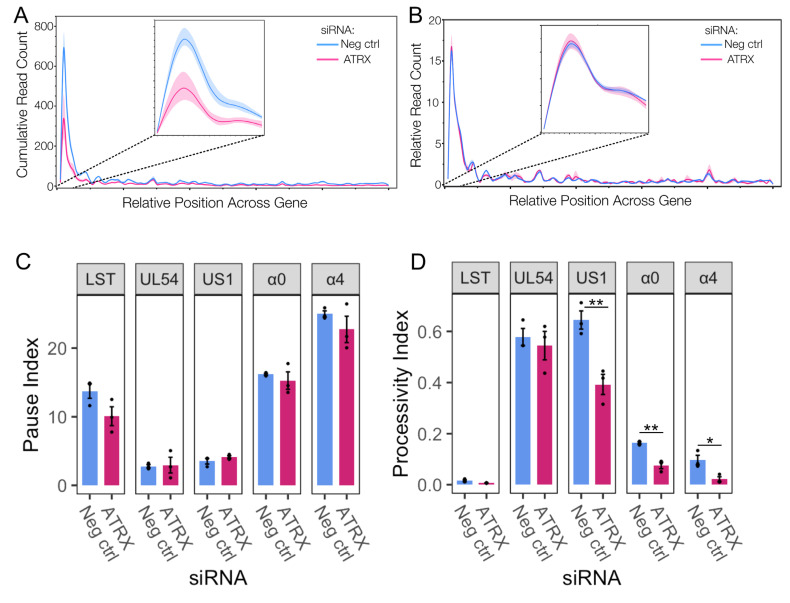
**ATRX functions through promoting initiation and does not affect Pol II pausing**. (**A**) Metaplot spline interpolation of cumulative PRO-Seq read count across HSV-IE genes during ATRX knockdown at 1.5 hpi. (**B**) Metaplot spline interpolation of relative read counts across HSV-IE genes during ATRX knockdown at 1.5 hpi. The bootstrap confidence of fit is shown in the shaded area. (**C**) Pause and processivity index values (**D**) of HSV-1 IE genes during ATRX knockdown at 1.5 hpi. Data are mean ± standard error. Statistical significance was determined using Welch’s *t*-test. Asterisks indicate statistical significance (* = *p* < 0.05, ** = *p* < 0.01).

Genome browser views of PRO-Seq data can obscure details due to the predominant peak near the promoter. Therefore, a metaplot of the cubic spline of PRO-Seq reads across IE genes was also plotted, highlighting the overall loss of transcriptional activity during ATRX depletion (Figure 4A). This reduction was apparent across the whole gene but was most striking at the promoter region (inset), the site of Pol II pausing. A reduction in a promoter peak is characteristic of a decrease in initiation [38], though it can also be due to an increased release of Pol II from pausing. To assess whether the loss of activity at the promoter was related to Pol II pausing and release, the relative activity across IE genes was calculated to measure the overall distribution of Pol II activity across genes, independent of read count. This revealed no difference in the ATRX KD compared to repair (Figure 4B), suggesting promoter-proximal pausing was unaltered. This was further confirmed by pause index analysis (ratio of read density in promoter v read density in gene body) on individual IE genes (Figure 4C).

We have previously shown that Pol II is highly processive across viral genes, and extensive transcription continues beyond the polyA site in a process tightly regulated by the virus [3,39]. A processivity index was calculated to quantify Pol II processivity (ratio of read density 1000 nt downstream of polyA site v read density in the gene body [39]). This revealed that specific IE genes (*US1, α0*, and *α4*) exhibit a reduction in Pol II processivity during ATRX depletion (Figure 4D), thus indicating a decrease in the amount of Pol II that successfully reaches and proceeds beyond the polyA site. In summary, these data suggest that ATRX promotes both transcription initiation and elongation efficiency of IE genes at 1.5 hpi.

### 3.4. ATRX Depletion Delays Progression Through the Temporal Cascade and Viral Replication

**Figure 5:**
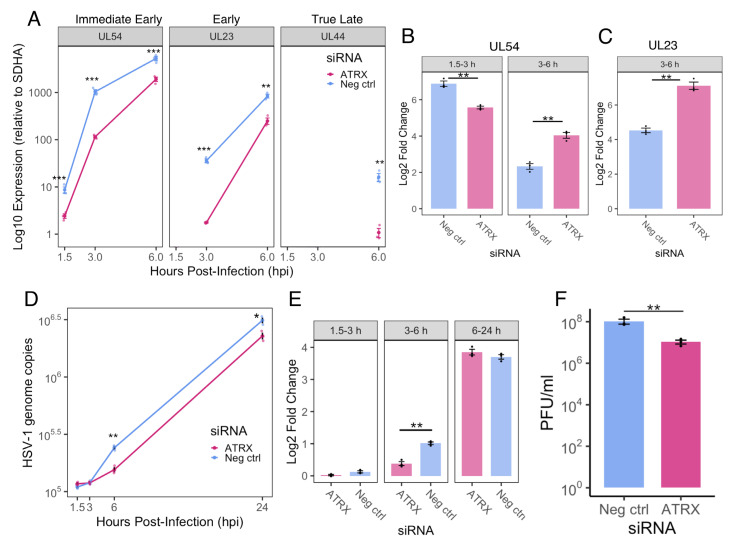
**ATRX KD delays progression through the HSV-1 replication cycle.** (**A**) Relative HSV-1 mRNA (normalized to SDHA) of genes three temporal classes at 1.5, 3 and 6 hpi. ND = not detected. Fold change in expression from 1.5–3 and 3–6 hpi for UL54 (**B**) and from 3–6 hpi for UL23 (**C**). (**D**) Time course of HSV-1 genome copy per 5 ng of DNA during siRNA treatment, determined by UL51 plasmid standard curve qPCR. (**E**) Fold change in HSV-1 genome copy from 1.5–3, 3–6 and 6–24 hpi. (**F**) HSV-1 titre at 24 hpi in siRNA treated cells. Data are mean ± standard error. Statistical significance was determined using Welch’s *t*-test. Asterisks indicate statistical significance (* = *p* < 0.05, ** = *p* < 0.01, *** = *p* < 0.001).

Next, we investigated whether the reduction in IE gene transcription efficiency during ATRX depletion impacted viral replication. First, we quantified mRNA levels of genes from different temporal classes using RT-qPCR over a time course of early infection. Transcript abundance was measured for an IE gene (*UL54*), an E gene (*UL23*), and a TL gene (*UL44*) at 1.5, 3, and 6 hpi. Across the time course, transcript levels for all genes were significantly reduced in ATRX-depleted cells compared to the negative control (Figure 5A). Despite overall lower transcript levels in ATRX KD, the rate of mRNA accumulation between 3–6 hpi was significantly higher than in controls for both *UL54* and *UL23* (Figure 5B,C). *UL44* could not be assessed in this analysis as it was undetectable at 3 hpi. These findings suggest that ATRX depletion primarily delays the onset of the transcriptional cascade by impairing IE gene expression. However, once the cascade progresses to E gene expression, transcription of these later gene subsets appears to accelerate.

We then assessed viral DNA replication by quantifying HSV-1 genomes using *UL51* qPCR at 1.5, 3, 6, and 24 hpi. ATRX depletion led to a modest (~1.5-fold) but significant reduction in viral genome copies at 6 and 24 hpi compared to the negative control (Figure 5D). Notably, from 3 to 6 hpi, the fold increase in genome copies was significantly greater in the negative control, whereas no difference was observed between conditions from 6 to 24 hpi (Figure 5E). These results suggest that ATRX does not directly impair viral DNA replication. Viral yield assays revealed an approximately 10-fold reduction in viral titre at 24 hpi in ATRX-depleted cells (Figure 5F). Taken together, these data indicate that reduced IE gene transcription efficiency during ATRX knockdown delays progression through the temporal cascade and replication cycle, resulting in a modest but significant impairment of viral progeny production.

### 3.5. ATRX Is Associated with Sites of Both Active and Repressed Transcription on the Cellular and Viral Genomes

The finding that ATRX promotes transcription on viral genes contrasts with its proposed role as a repressor. The repressive activity of ATRX is believed to result from its binding to the viral genome to promote repressive heterochromatin [13,20]. However, the high levels of transcriptional activity on the viral genome detected by this PRO-Seq study and others [3,5,30,39,40] is not characteristic of a heterochromatin state. Recent advancements in ChIP using ethylene glycol-bis(succinimidylsuccinate) (EGS) to preserve indirect and/or hyperdynamic interactions [21] have shown that ATRX is associated with both euchromatin and heterochromatin on the cellular genome [41]. We, therefore, chose to use this enhanced ChIP-Seq protocol for in-depth characterization of ATRX binding on the viral genome during lytic infection. HEp-2 cells were infected with HSV-1 at an MOI of 5 PFU/cell before EGS/formalin fixation, and ChIP was then performed.

To validate the success of the ChIP-Seq, we first examined ATRX binding at cellular sites. Peaks were called using MACS3 [33], and PRO-Seq data from an independent, non-siRNA-treated experiment was used to assess transcriptional activity at these sites. ATRX peaks were identified in both transcriptionally active and repressed regions, consistent with previous EGS ATRX-ChIP studies [41] (Figure S4). A full list of peak binding sites is provided in Supplementary Data S1.

**Figure 6:**
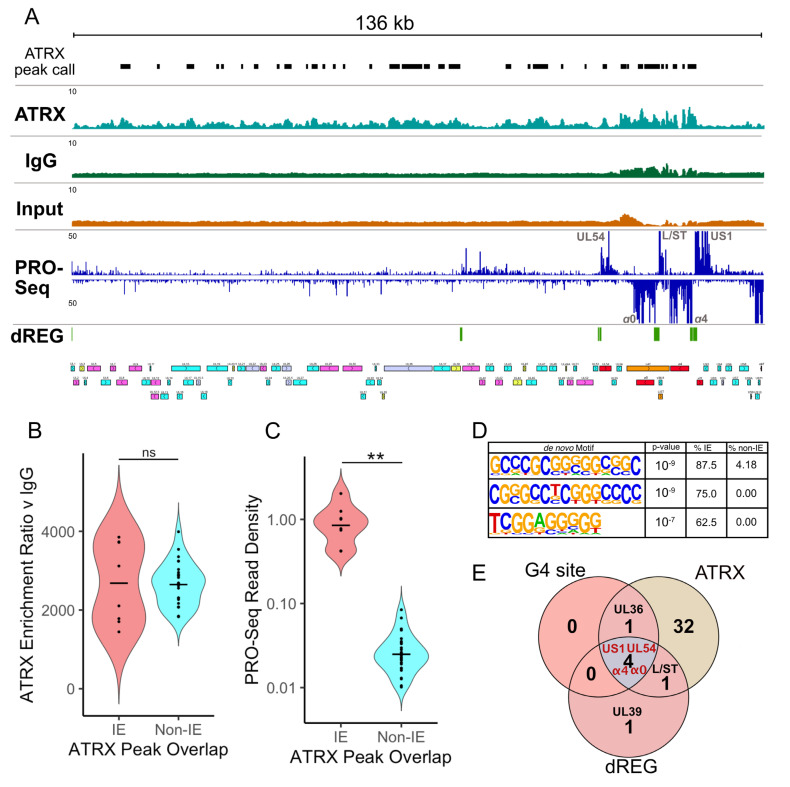
**ATRX is associated with active and repressed transcription sites on the viral genome.** (**A**) Genome browser view of ATRX ChIP-Seq data across the HSV-1 F genome at 1.5 hpi with MACS3 peak calls detailed (mean of 3 replicates). HSV-1 ChIP-Seq coverage files were normalized to HSV-1 RPGC (reads per genomic content). PRO-Seq data (mean of 2 replicates) is also shown, with dREG identification of transcriptionally regulatory regions. (**B**) MACS3 likelihood enrichment ratio of ATRX peaks, split by whether they overlap IE or non-IE genes. (**C**) PRO-Seq read density at ATRX peaks, split by whether they overlap IE or non-IE genes. The black line represents the mean. Statistical significance was determined using the Wilcoxon test. Asterisks indicate statistical significance (** = *p* < 0.01, ns = non-significant). (**D**) Enriched motifs in ATRX peaks that overlap IE genes, relative to non-IE genes. (**E**) Overlap between viral genes, which contain G-quadruplex (G4) sites, ATRX peak, and active transcription (dREG).

Having validated this approach for detecting ATRX binding, we next aligned the data to the HSV-1 genome (Figure 6A). As the viral genome is primarily free of tightly bound histones during lytic infection [8,42,43], there is the potential for unspecific IgG pulldown of viral DNA. Therefore, a stringent two-step MACS3 analysis was performed in which peaks were initially called for ATRX v Input and IgG v Input. Differential peak analysis was then applied, using a likelihood ratio to identify ATRX peaks enriched relative to IgG. The dREG tool was used on the PRO-Seq data to determine transcriptional regulatory elements (TREs) associated with accessible chromatin [44]. This revealed that ATRX was enriched across the viral genome at 1.5 hpi at both highly transcriptionally active and accessible IE genes as well as transcriptionally restricted sections of the genome (Figure 6A). To quantify these observations, ATRX binding peaks were categorized according to whether they overlapped IE or non-IE genes. This confirmed no difference in ATRX enrichment between IE and non-IE genes (Figure 6B); however, the PRO-Seq read density in ATRX peaks was significantly higher for those that overlapped IE genes (Figure 6C). Overall, this analysis indicated ATRX binding on the viral genome during early lytic infection is associated with both highly active and restricted transcription.

This differential activity in transcription at ATRX binding sites suggests there are genomic features that might impact ATRX function. To investigate this possibility, HOMER [34] motif enrichment was used to identify sequence motifs enriched in ATRX peaks at transcriptionally active (IE) v transcriptionally restricted (non-IE) regions. Interestingly, the top three enriched motifs contained repetitive G-rich tracts (Figure 6D), characteristic of G4 forming sequences [45]. This was intriguing because ATRX was previously shown to affect gene expression through interaction with G4 regions on the cellular genome [41,46]. In addition, the HSV-1 genome contains multiple validated G4 sites, including in the promoters of IE genes [23,24]. The link between active transcription (dREG), G4s, and ATRX binding was highlighted when viral genes that contain these features were overlapped, revealing that IE genes (*UL54*, *α0*, *α4*, and *US1*) contain all three features (Figure 6E).

### 3.6. Stabilization of G-Quadruplexes Mimics the Effects of ATRX Depletion on Viral Transcription

The finding that ATRX binding on the highly transcriptional active IE genes was also linked to the presence of G4s raised the hypothesis that ATRX depletion alters G4 formation on the viral genome, thereby restricting transcription. To investigate this, we utilized the G4-ligand, BRACO-19 [47], to stabilize G4 formation during viral infection. First, we tested nascent transcription using the PRO-qPCR method. HFF and HEp-2 cells were infected with HSV-1 at an MOI of 5 PFU/cell. After a 1 h absorption period at 4 °C, infection was allowed to proceed at 37 °C in media containing either 25 μM BRACO-19 or DMSO. Nuclei were harvested at 1.5 hpi, and PRO-RTqPCR performed. This indicated a loss of nascent viral transcription during BRACO-19 treatment in both HEp-2 and HFF cells (Figure S3).

**Figure 7:**
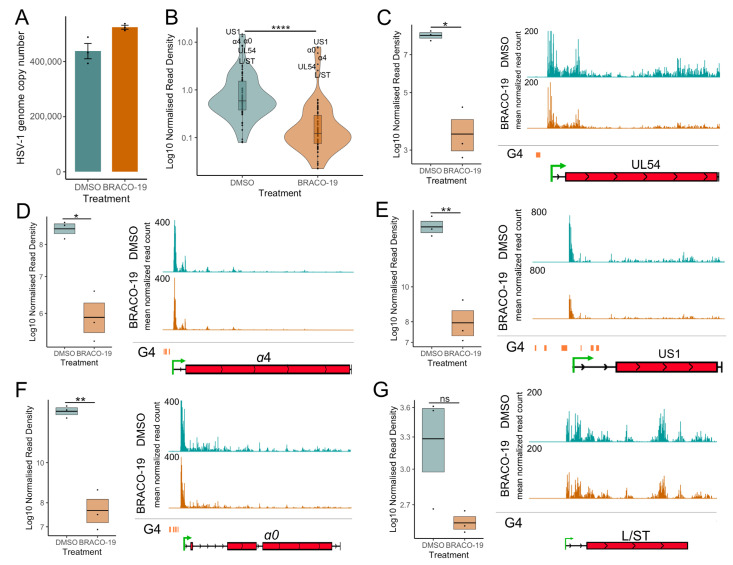
**Stabilization of G-quadruplexes reduces transcriptional activity across HSV-1 genes**. (**A**) HSV-1 genome copy per 5 ng of DNA in BRACO-19 or DMSO treatment at 1.5 hpi, determined by UL51 plasmid standard curve qPCR. Data are mean ± standard error. (**B**) PRO-Seq read density across viral genes at 1.5 hpi during BRACO-19 treatment, compared to DMSO treatment. Statistical significance was determined using the Wilcoxon test. PRO-Seq read density of individual IE genes and genome browser tracks at 1.5 hpi of (**C**) UL54, (**D**) α4, (**E**) US1, (**F**) α0 and (**G**) L/ST. PRO-Seq read density is calculated as read per bp, normalized to spike-in and HSV-1 genome copy. Black lines indicate mean and bands ± standard error. Statistical significance was determined using Welch’s *t*-test. Asterisks indicate statistical significance (* = *p* < 0.05, ** = *p* < 0.01, **** = *p* < 0.0001, ns = non-significant).

Next, we used PRO-Seq to analyze nascent transcription during BRACO-19 treatment in HEp-2 cells. Infections and drug treatments were performed as described above; nuclei were harvested at 1.5 hpi, and PRO-Seq was performed. qPCR was used to confirm the treatment did not affect infectivity, revealing only a slight variation in viral genome copy number between treatments (Figure 7A). These data were included in the PRO-Seq normalization (details in Table S3). Treatment of cells with BRACO-19 led to a significant reduction in transcriptional activity across all viral genes at 1.5 hpi (Figure 7B), similar to ATRX KD (Figure 3C). Focusing our analysis on IE genes confirmed a significant reduction in PRO-Seq read density across all IE genes, which was also evident through IGV genome browser visualization of PRO-Seq data (Figure 7D–F). As with ATRX KD, transcription of *L/ST* (which does not contain a G4 in its promoter) was not significantly affected by BRACO-19 treatment (Figure 7G).

**Figure 8:**
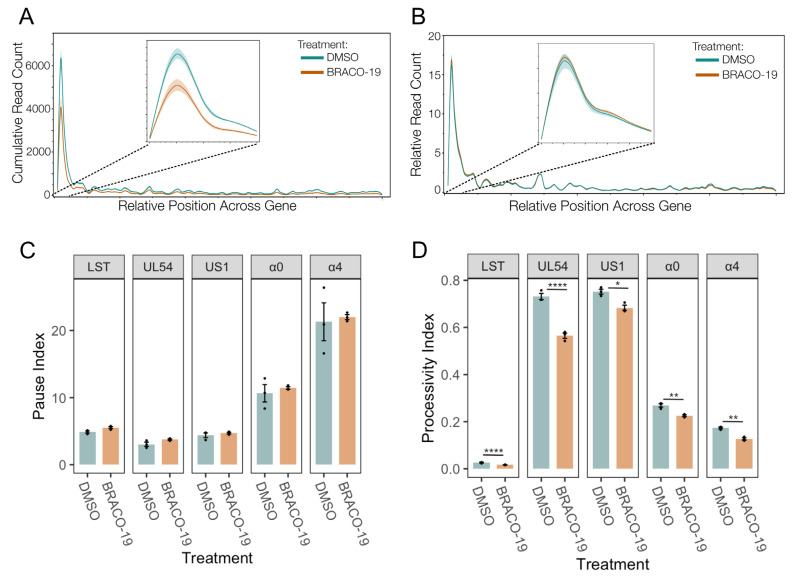
**Stabilization of G-quadruplexes reduces transcriptional initiation and does not affect Pol II pausing.** (**A**) Metaplot spline interpolation of cumulative PRO-Seq read count across HSV-IE genes during BRACO-19 treatment at 1.5 hpi. (**B**) Metaplot spline interpolation of relative read counts across HSV-IE genes during BRACO-19 treatment at 1.5 hpi. The bootstrap confidence of fit is shown in the shaded area. (**C**) Pause and processivity index values (**D**) of HSV-1 IE genes during BRACO-19 treatment at 1.5 hpi. Data are mean ± standard error. Statistical significance was determined using Welch’s *t*-test. Asterisks indicate statistical significance (* = *p* < 0.05, ** = *p* < 0.01, **** = *p* < 0.0001).

A metaplot of the cubic spline of the reads across IE genes was generated to quantify and compare the effect on promoter activity. This confirmed a significant reduction of transcriptional activity at IE gene promoters after BRACO-19 treatment (Figure 8A), suggesting decreased initiation. To assess the potential for this promoter-proximal loss of activity to be a result of pause release, the relative distribution of reads was also plotted (Figure 8B). This revealed no difference in relative transcriptional activity between BRACO-19 and DMSO treatment, indicating the effect at the promoter was not due to accelerated pause release. This was confirmed by the calculation of pause indices for all IE genes, all of which were unaffected by BRACO-19 treatment (Figure 8C). Processivity index calculation revealed a significant reduction in Pol II processivity across all IE genes after BRACO-19 treatment (Figure 8D), suggesting G4 stabilization restricts both initiation and efficient elongation, again similar to ATRX depletion (Figure 4D).

**Figure 9:**
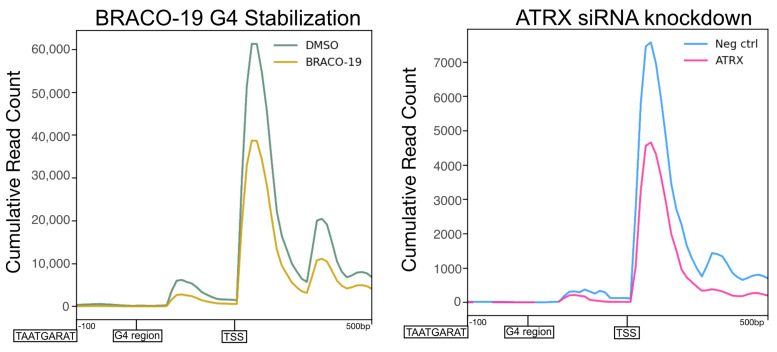
**Stabilization of G-quadruplexes mimics the effects of ATRX depletion on viral transcription**. Profile of PRO-Seq reads at −100–+500 G4 regions in the promoters of HSV-1 IE genes at 1.5 hpi during BRACO-19 treatment compared to DMSO treatment ATRX knockdown compared to neg ctrl (non-targeting) knockdown.

Overall, it was apparent that BRACO-19 stabilization of G4 quadruplexes mimicked the effects of ATRX KD on IE gene transcription, with decreased initiation as the predominant effect. A summary of this is shown in Figure 9, in which the density of PRO-Seq reads was plotted centered on IE gene promoter G4 regions. The profile of PRO-Seq reads after depletion of ATRX or BRACO-19 stabilization were almost identical at these regions, with a significant reduction in transcription at the TSS downstream of the G4 region.

## 4. Discussion

We had hypothesized that PML-NB components contribute to the transient repression of HSV-1IE genes described in our TIEMR model. Using PRO-Seq, which provides nucleotide-resolution insight into nascent transcription, we unexpectedly found that one such component, ATRX, promotes rather than represses transcription initiation at IE promoters. This activity appears specific to initiation, as Pol II pausing was unaffected by ATRX depletion (Figure 4A,B). We also observed reduced Pol II processivity across select IE genes in ATRX-depleted cells, consistent with earlier reports that diminished initiation leads to impaired elongation on HSV-1 genes, potentially due to decreased accessibility and reduced recruitment of elongation factors. These findings suggest that while PML-NBs act as an initial site of genome entrapment and repression, ATRX may paradoxically facilitate escape from TIEMR by promoting IE transcription initiation.

Our ChIP-Seq data showed ATRX associates with both active and inactive viral loci, mirroring its diverse roles on the cellular genome. A key feature of genomic regions with ATRX binding at active transcription is the presence of G-rich sequences, characteristic of G4-forming motifs [45]. The HSV-1 genome contains multiple validated G4 sites [23,48], including those at promoters of the highly transcriptionally active IE genes [24]. Destabilization of these G4s has been shown to promote IE gene expression [25]. Interestingly, ATRX depletion alters the expression of cellular genes with G4 motifs in their promoters [41,46], a phenomenon thought to result from increased G4 formation [46,49].

We hypothesized that ATRX may function similarly on the viral genome by facilitating G4 destabilization and that ligand-induced stabilization of G4s would mimic ATRX loss. Consistent with this model, treatment with the G4-stabilizing ligand, BRACO-19 (also known to exhibit antiviral activity by reducing viral DNA polymerase processivity [23]), phenocopied ATRX depletion. Specifically, G4 stabilization led to a comparable reduction in transcriptional initiation of IE genes (Figure 9). Notably, transcription of *L/ST* was unaffected by either ATRX depletion or BRACO-19 treatment. As the *L/ST* promoter lacks a VP16 transactivation motif (TAATGARAT) and G4 elements, it is probable that ATRX’s function is linked to the distinct promoter architecture of IE genes. G4 stabilization reduces transcriptional initiation and subsequent nascent RNA synthesis of cellular genes due to impaired loading of general transcription factors, including TATA-binding protein (TBP) [50]. Since most HSV-1 promoters contain TATA boxes and TBP is associated with IE promoters [43,51], our model proposes that enhanced G4 formation, presumably occurring in the absence of ATRX, hinders the recruitment of TBP and other factors necessary for efficient transcription.

Although ATRX knockdown increases G4 formation on cellular DNA [49,52,53], directly confirming a similar effect on the viral genome is experimentally challenging. Prior studies primarily relied on immunofluorescence to quantify G4 formation, which cannot distinguish between viral and cellular DNA. However, the striking phenotypic similarity between ATRX depletion and G4 stabilization with respect to impaired viral transcriptional initiation strongly suggests a mechanistic link between ATRX and G4s. Nonetheless, this connection remains to be fully validated experimentally and represents a limitation of the current study.

ATRX contains an ATPase/helicase domain and is a member of the SWI/SNF family of chromatin remodeling proteins, so its involvement in regulating DNA accessibility and transcription is not unexpected [54]. However, the pro-transcriptional role of ATRX on the HSV-1 genome observed here was surprising, given that previous studies have primarily suggested a repressive function [13,36,55,56]. Notably, much of that evidence comes from experiments using ICP0-null viruses, and ATRX was thought to have little impact on wild-type HSV-1 [36]. To our knowledge, this is the first study to assess the effect of ATRX depletion on early viral nascent transcription using high-resolution transcriptomics. Prior studies relied on mRNA or protein measurements at later time points, which do not necessarily reflect early nascent transcriptional activity or kinetics. Knockdown strategies can also yield variable outcomes [16], and the use of constitutive knockout cell lines in other studies may partly explain the conflicting findings. We acknowledge the limitation of using a single siRNA approach. However, the increase in nascent transcription following DAXX knockdown supports the validity of our transient siRNA system and aligns with its known repressive role [10,16,36]. Importantly, other PML-NB components remained stable (Figure 2), and input viral genome levels were consistent across conditions, suggesting the phenotype is attributable to ATRX knockdown rather than indirect effects.

ATRX has been proposed to repress transcription by stabilizing H3.3 on the viral genome, promoting heterochromatin formation [13,20]. While this may support latency, in which viral DNA is assembled into nucleosomal chromatin and transcription is restricted [57,58], chromatin organization is less ordered during lytic infection [8,42,43,59], in which the viral chromatin structure is heterogeneous [60,61]. Histone association with DNA also does not necessarily confer nucleosome formation, a key feature of heterochromatin. Indeed, ATAC-Seq studies have revealed that the viral genome is highly accessible during lytic infection, with no evidence of nucleosome-mediated protection [43,56]. PRO-Seq studies also confirm that the viral genome remains highly transcriptionally active throughout lytic infection [3,5,30,39].

Together, these findings suggest that the viral genome is not incorporated into heterochromatin during early lytic infection. Although H3.3 is deposited on the viral genome during this stage [13,61,62], this histone variant is also enriched at active, accessible chromatin regions on the cellular genome, particularly promoters and enhancers [63,64]. H3.3 incorporation is also known to promote transcription on the HSV-1 genome [62,65], indicating it is not exclusively associated with transcriptional repression. Furthermore, ATRX knockout does not significantly affect viral genome accessibility in the first 4 h of infection [56]. These observations support a model in which ATRX stabilization of H3.3 on the viral genome may promote transcription, rather than repress through heterochromatin formation.

As on the cellular genome, ATRX likely plays complex, context-dependent roles in regulating viral gene expression. We propose that ATRX acts in a pro-transcriptional capacity early in infection, when the genome is largely nucleosome-free, but may adopt a repressive function as the genome becomes more chromatinized. Supporting this, we observed that although ATRX depletion reduced overall mRNA and DNA accumulation (Figure 5), the subsequent rate of production of mRNA/DNA after 3 hpi was significantly higher than in control. Thus, ATRX may contribute to transcriptional repression during later stages of lytic infection as previously reported [13,20,56,66]. This also aligns with its known role in maintaining transcriptional repression during latency in neurons, when the viral genome is enriched for repressive histone marks such as H3K9me3 (20, 50). These observations suggest that the mechanisms underlying TIEMR and ATRX’s role in this process depend on both the genome and the cellular context. TIEMR and pro-transcriptional ATRX function likely occurs on newly delivered or freshly replicated viral DNA, whereas its function shifts toward repression on the nucleosomally repressed genome during latency, especially as ATRX is highly abundant in neurons [67].

Incorporation of our data with previous studies supports a model in which incoming HSV-1 genomes are rapidly entrapped and repressed by PML-NBs. Rather than being purely defensive, this repression may benefit the virus by promoting TIEMR to help coordinate the subsequent transcriptional cascade. As viral genomes lack nucleosomes early in infection [8,43,61,68], this repression likely involves DNA compaction rather than canonical heterochromatin formation. Our data indicate that DAXX and ATRX have distinct roles in regulating the early transcription of PML-NB-entrapped genomes: DAXX promotes repression, which is alleviated only when sufficient levels of ICP0 are expressed to displace it. ATRX appears to facilitate IE gene transcription (and thus ICP0 expression) by interacting with G4-containing IE promoters, enabling the virus to proceed with de-repression and progression through the transcriptional cascade.

Collectively, our findings highlight the complexity of HSV-1’s transcriptional regulation, shaped over millions of years of co-evolution with humans [69]. The virus must balance repression—minimizing immune detection—with activation, ensuring that a subset of genomes initiate productive infection. This finely tuned regulation is likely essential in neurons, where the virus must preserve host cell viability to establish and maintain latency.

## Data Availability

The data is publicly available on the GEO database under the accession numbers: GSE293672, GSE293673, GSE293674 and GSE293675.

## Supplementary Materials

The following supporting information can be downloaded at: https://www.mdpi.com/article/10.3390/v17091169/s1, Figure S1: PRO-qPCR during ATRX and DAXX KD in HFF cells. Figure S2: ATRX and DAXX mRNA expression during ATRX and DAXX KD. Figure S3: PRO-qPCR during DMSO/BRACO-19 treatment in HEp-2 and HFF cells. Figure S4: EGS ATRX-ChIP cellular results. Table S1: primers used in this study. Table S2: Antibodies used in this study. Table S3: PRO-Seq Normalisation details. Supplementary Data S1: Details of ATRX ChIP-Seq peaks.
