# Supplementary material for: ATRX Promotes Transcription Initiation of HSV-1 Immediate Early Genes During Early Lytic Infection
Source: Viruses. 2025 Aug 27;17(9):1169. doi: 10.3390/v17091169 (PMC12474073; doi:10.3390/v17091169)
Supplement: Supplementary file 1 [file viruses-17-01169-s001.zip › Suppplementaty Table S1-S3.pdf]

Table S1: Primers

Gene	Forward (5’-3’)	Reverse (5’-3’)	Target
**PML**	CCGTCATAGGAAGTGAGG CTTC	GTTCGCATCTGAGTCTCCG	mRNA
**ATRX**	ACGGCGTAGTGGTTGTCTC	GCAGCATGTAGCCCTCT	mRNA
**DAXX**	GAAGCTCTGGATTCTGTG	CATCACTCTCTCATCGTCTCG	mRNA
**SDHA**	GGACAACTGGAGGTGGCATT	CCGTCATGTAGTGGATGGCA	mRNA
**UL23**	ACCGCTAACAGCGTCAACA	CAAAGAGGTGCGGGAGTT	mRNA
**UL44**	GTGACGTTTGCCTGGTTCCTGG	GCACGACTCCTGGCCGTACG	mRNA
**UL2**	CTGTGAAGGCTGGGTGTG	GTAGT CAAATCTGTCGC	Nascent RNA/mRNA
**UL54**	GCATCCTTCGTGTTTGTCATTCTG	GCATCTTCTCTCCGACCCCG	Nascent RNA/mRNA
**US1**	TTTGGGGAGTTTGACTGGAC	CAGACACTTGCGGTCTTCTG	Nascent RNA/mRNA
**ACTB**	GCTCATGTAGAAGGTGTG	GCATGGTCAGAAGGATCT	Nascent RNA
**UL51**	GCCAGTCGTTCTAGGTTCAC	GTTAACGCGCTACTTCCCG	HSV-1 DNA

Table S2: Antibodies

Antibody/Stain	Source	Use
**ATRX (Rabbit Polyclonal)**	Bethyl (#A301-045A)	IF (1:100)
**DAXX (Rabbit Polyclonal)**	MilliporeSigma (#07-471)	IF (1:100)
**PML (Mouse Monoclonal)**	Abcam (#ab9605)	IF (1:500)
**Donkey Anti-rabbit IgG Alexafluor 568**	Invitrogen (#A10042)	IF (1:500)
**Donkey Anti-mouse IgG Alexafluor 647**	Invitrogen (#31571)	IF (1:500)
**DAPI**	Invitrogen (#D1306)	IF (1μg/ml)
**ATRX (Rabbit Polyclonal)**	Abcam (#ab97508)	ChIP (10μg per IP)
**IgG (Rabbit Polyclonal)**	Abcam (#ab171870)	ChIP (10μg per IP)

Table S3: PRO-Seq Normalization Details

ATRX KD PRO-Seq

Sample	*Total Paired Reads*	*Library correction factor*	*HSV1 genome copies (qPCR)*	*Genome copy correction factor*	*Combined correction factor*
Neg_KD_1	12664906	1.12	382102.09	0.98	1.09
Neg_KD_2	13876038	1.02	352869.89	1.06	1.08
Neg_KD_3	11975852	1.18	280353.42	1.33	1.57
ATRX_KD_1	12083661	1.17	371827.00	1.00	1.18
ATRX_KD_2	15752967	0.90	448850.98	0.83	0.75
ATRX_KD_3	18534405	0.76	403396.05	0.93	0.71
**MEAN**	14147972	**MEAN:**	373233.24		

HEp-2 1.5 hpi PRO-Seq (for ATRX ChIP-Seq comparison)

Sample	*Total Paired Reads*	*Library correction factor*	*HSV1 genome copies (qPCR)*	*Genome copy correction factor*	*Combined correction factor*
Hep2_1	64737291	1.07	26449.9021	1.02	1.09
Hep2_2	74188010	0.94	27429.3679	0.98	0.92
**MEAN**	69462650.5	**MEAN**	26939.635		

BRACO-19 PRO-Seq

Sample	*Total Paired Reads*	*Library correction factor*	*HSV1 genome copies (qPCR)*	*Genome copy correction factor*	*Combined correction factor*
DMSO_1	63318485	1.06	488329.607	0.99	1.04
DMSO_2	68306699	0.98	431937.951	1.11	1.09
DMSO_3	59719519	1.12	393094.074	1.22	1.37
BRACO19_1	66106799	1.01	523448.398	0.92	0.93
BRACO19_2	78368896	0.86	512761.915	0.94	0.80
BRACO19_3	66530182	1.01	537273.125	0.90	0.90
**MEAN**	67058430	**MEAN:**	481140.845		
