# Supplementary figures and images for: ATRX Promotes Transcription Initiation of HSV-1 Immediate Early Genes During Early Lytic Infection
Source: Viruses. 2025 Aug 27;17(9):1169. doi: 10.3390/v17091169 (PMC12474073; doi:10.3390/v17091169)

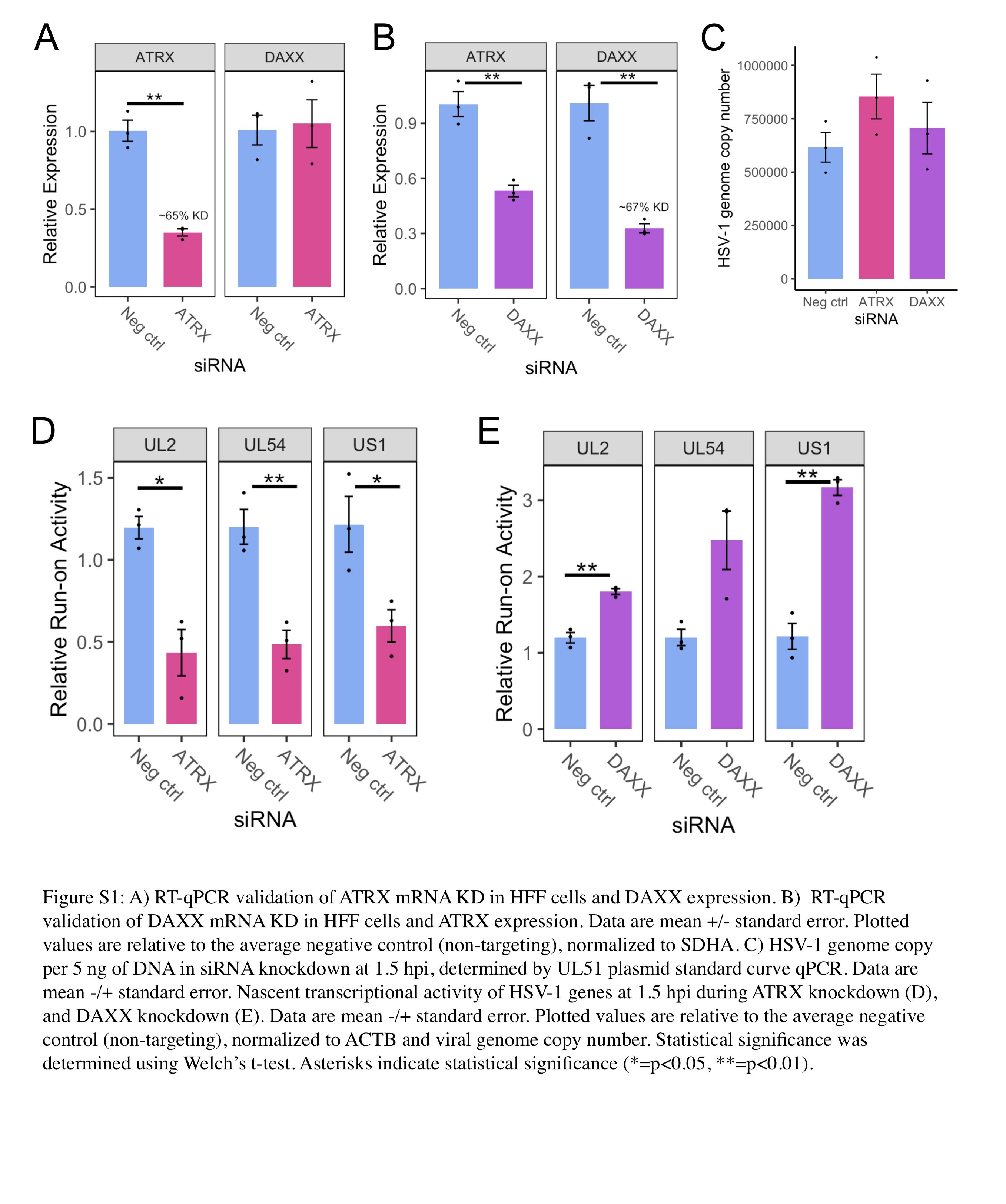

Supplement: Supplementary file 1 [file viruses-17-01169-s001.zip › Figure_S1.tiff]

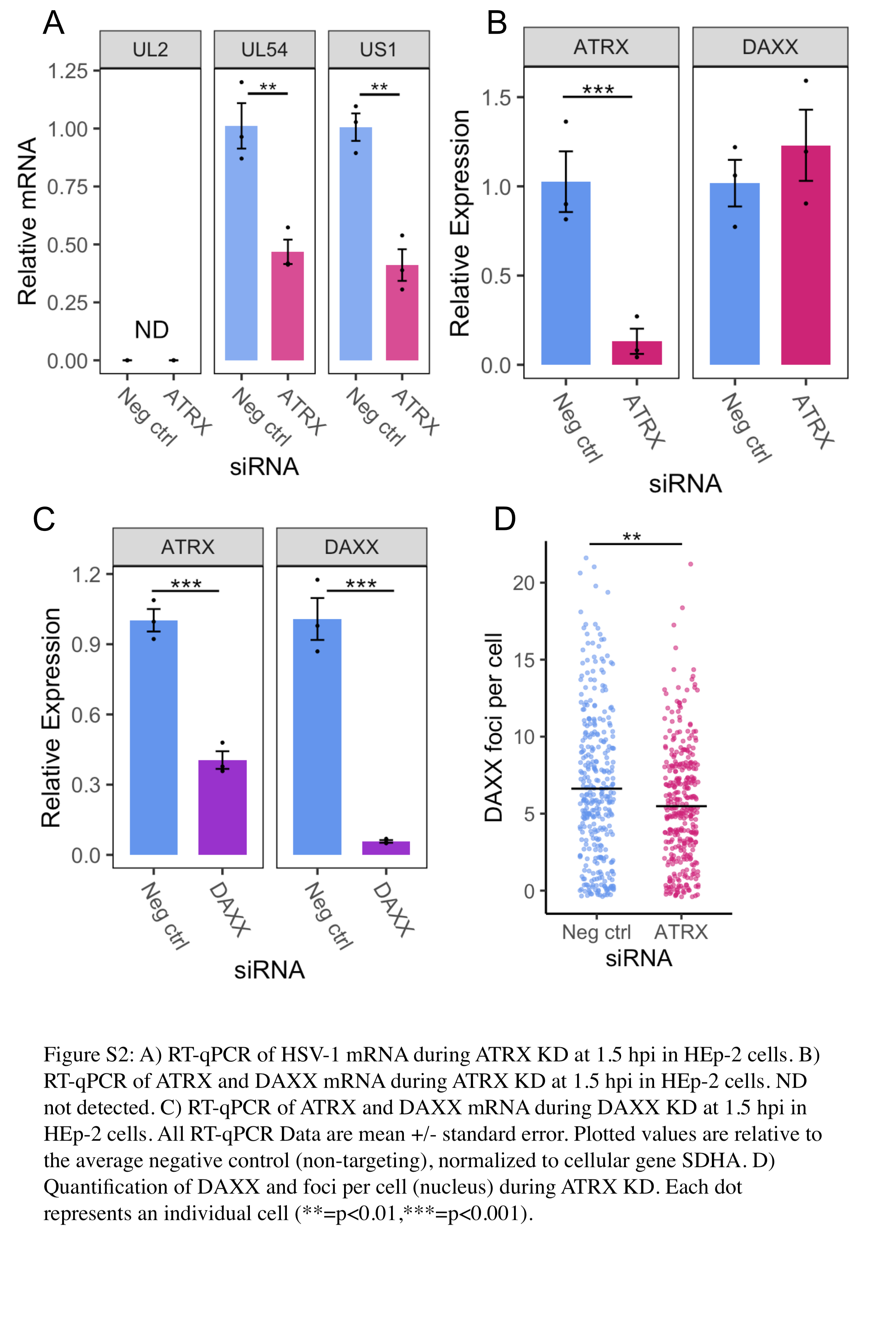

Supplement: Supplementary file 1 [file viruses-17-01169-s001.zip › Figure_S2.tiff]

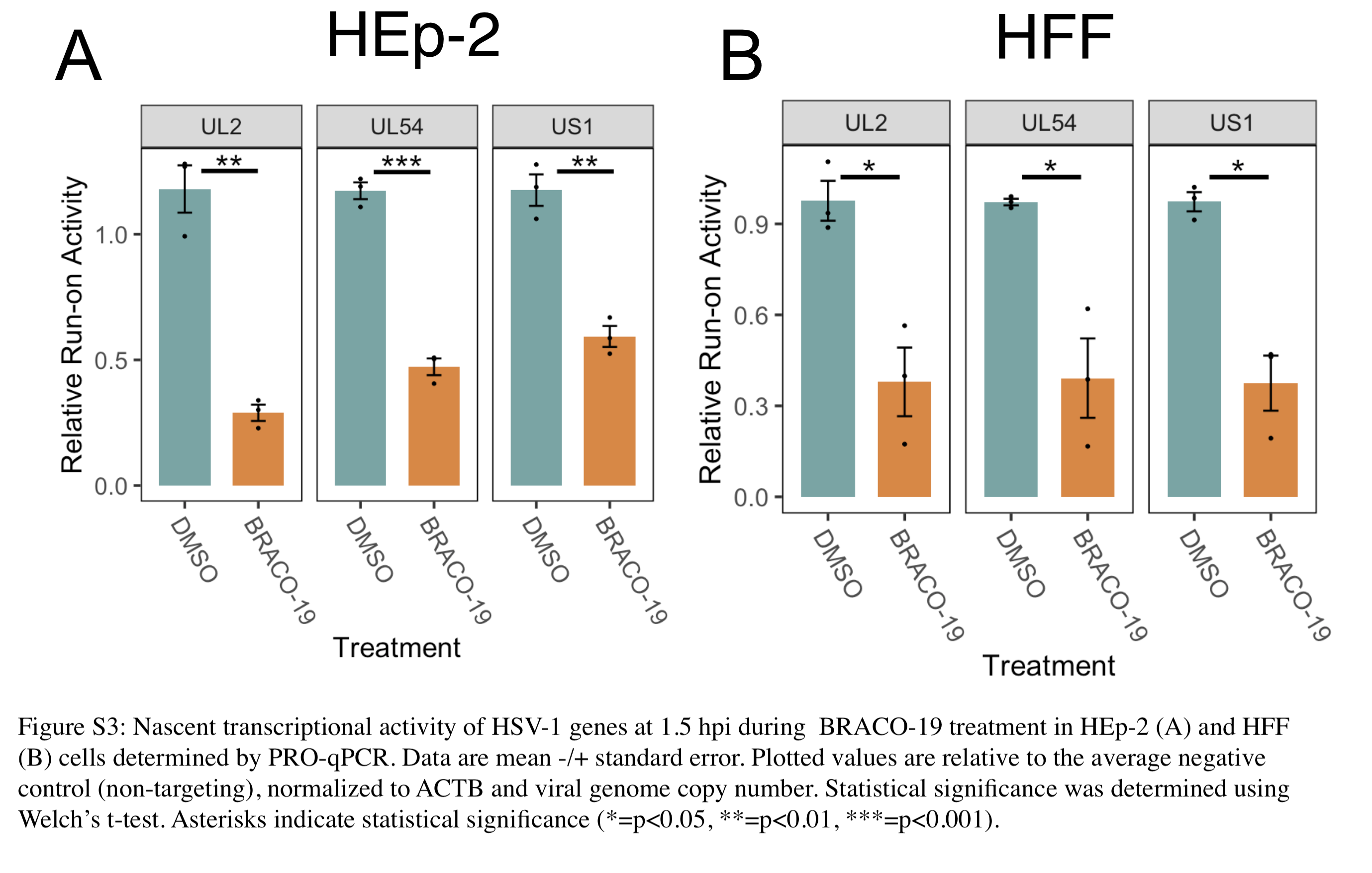

Supplement: Supplementary file 1 [file viruses-17-01169-s001.zip › Figure_S3.tiff]

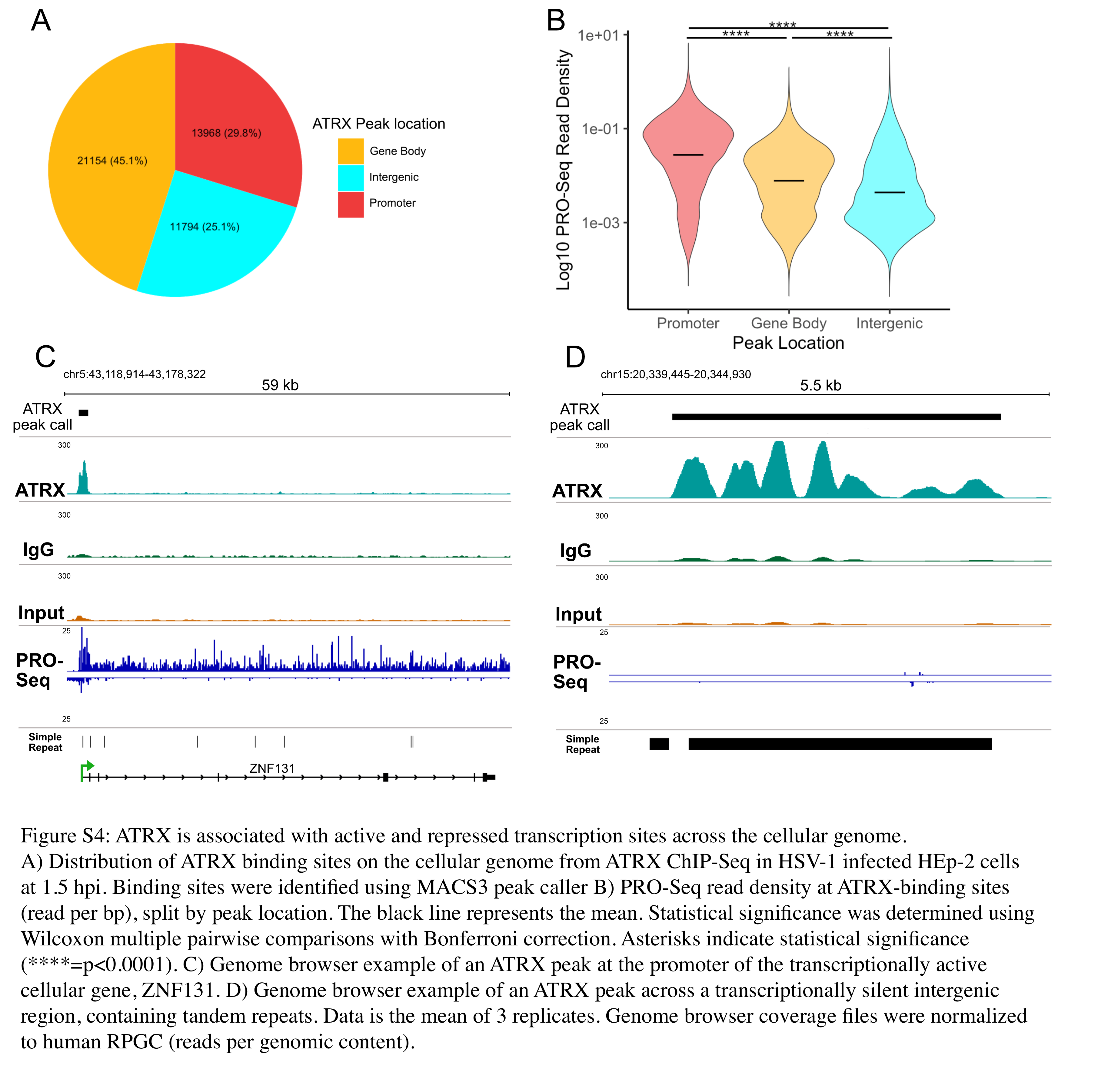

Supplement: Supplementary file 1 [file viruses-17-01169-s001.zip › Figure_S4.tiff]
